# *quatre-quart1* is an indispensable U12 intron-containing gene that plays a crucial role in Arabidopsis development

**DOI:** 10.1093/jxb/erx138

**Published:** 2017-05-05

**Authors:** Kyung Jin Kwak, Bo Mi Kim, Kwanuk Lee, Hunseung Kang

**Affiliations:** Department of Plant Biotechnology, College of Agriculture and Life Sciences, Chonnam National University, Gwangju, Korea

**Keywords:** *Arabidopsis thaliana*, development, intron, *quatre-quart (QQT1*), splicing, U12 intron

## Abstract

Despite increasing understanding of the importance of the splicing of U12-type introns in plant development, the key question of which U12 intron-containing genes are essential for plant development has not yet been explored. Here, we assessed the functional role of the *quatre-quart1* (*QQT1*) gene, one of the ~230 U12 intron-containing genes in *Arabidopsis thaliana*. Expression of *QQT1* in the U11/U12-31K small nuclear ribonucleoprotein mutant (*31k*) rescued the developmental-defect phenotypes of the *31k* mutant, whereas the miRNA-mediated *qqt1* knockdown mutants displayed severe defects in growth and development, including severely arrested stem growth, small size, and the formation of serrated leaves. The structures of the shoot apical meristems in the *qqt1* mutants were abnormal and disordered. Identification of QQT1-interacting proteins via a yeast two-hybrid screening and a firefly luciferase complementation-imaging assay revealed that a variety of proteins, including many chloroplast-targeted proteins, interacted with QQT1. Importantly, the levels of chloroplast-targeted proteins in the chloroplast were reduced, and the chloroplast structure was abnormal in the *qqt1* mutant. Collectively, these results provide clear evidence that *QQT1* is an indispensable U12 intron-containing gene whose correct splicing is crucial for the normal development of Arabidopsis.

## Teaser

*quatre-quart1* is an indispensable U12 intron-containing gene, and correct splicing of the U12 intron in *quatre-quart1* is crucial for the normal development of *Arabidopsis thaliana*.

## Introduction

Splicing of introns in precursor mRNAs is essential for the regulation of gene expression in eukaryotes. There are two types of introns: U2-type major introns and U12-type minor introns (Moore and Sharpe, 1993; Staley and Guthrie, 1998). Although the U12-type introns are significantly less frequent than the U2-type introns, constituting <1% of introns found in animals and plants (Levine and Durbin, 2001; Zhu and Brendel, 2003; Alioto, 2007), they play a substantial role in many organisms. The importance of U12 intron splicing has been demonstrated in the development of animals (Otake *et al.*, 2002; Edery *et al.*, 2011; He *et al.*, 2011; Sikand and Shukla, 2011) and in nonsense-mediated mRNA decay and cell viability (Hirose *et al.*, 2004; Will *et al.*, 2004). During the assembly and function of the minor spliceosome, the U11 and U12 small nuclear ribonucleoproteins (snRNPs) form a U11/U12 di-snRNP complex, and several proteins, including U11/U12-20K, -25K, -31K, -35K, -48K, -59K, and -65K, are uniquely associated with the minor spliceosome in both animals and plants (Schneider *et al.*, 2002; Will *et al.*, 2004). Furthermore, these minor spliceosome-specific proteins are well conserved in both dicot and monocot plants (Schneider *et al.*, 2002; Will *et al.*, 2004; Russell *et al.*, 2006).

The Arabidopsis genome harbors ~230 U12 intron-containing genes (Alioto, 2007; Jung and Kang, 2014), and several previous studies have shown that Arabidopsis U11/U12-31K, U11-48K, and U11/U12-65K are indispensable for the correct splicing of most of the U12 intron-containing genes, which is crucial for the normal development of *Arabidopsis thaliana* (Kim *et al.*, 2010; Kwak *et al.*, 2012; Jung and Kang, 2014; Xu *et al.*, 2016). Although these previous studies clearly demonstrated that the proper splicing of U12-type introns is essential for plant development, the key question of which U12 intron-containing genes are essential for normal Arabidopsis development has not yet been explored. Moreover, given that more than half of the putative U12 intron-containing genes encode proteins with unknown biological functions, it is currently not possible to establish a clear relationship between the developmental-defect phenotypes and abnormal splicing of specific U12 introns (Kim *et al.*, 2010; Jung and Kang, 2014).

*quatre-quart1* (*QQT1*; At5g22370) is an U12 intron-containing gene that encodes an ATP/GTP-binding protein. It was demonstrated that mutation in *QQT1* or in *QQT2* (At4g21800) results in embryo-defective phenotypes with embryos arrested at the octant stage, suggesting that *QQT1* and *QQT2* are essential for early embryo development (Lahmy *et al.*, 2007). Our previous study demonstrated that *QQT1* was one of the U12 intron-containing genes whose intron splicing was severely impaired in the U11/U12-31K mutant (*31k*) (Kim *et al.*, 2010). To determine whether the defect in U12 intron splicing of *QQT1* is closely correlated with the developmental defects of *A. thaliana*, we generated transgenic Arabidopsis plants that express *QQT1* in the *31k* mutant background as well as the artificial miRNA (amiR)-mediated knockdown mutants of *QQT1*, and analyzed their development-related phenotypes. We show clear evidence that *QQT1* is an indispensable U12 intron-containing gene that is crucial for the normal development of *A. thaliana*.

## Materials and methods

### Plant materials and growth conditions

*Arabidopsis thaliana* Columbia-0 ecotype was grown at 23 °C under long-day conditions (16 h light/8 h dark cycle). The *U11/U12-31K* knockdown Arabidopsis mutant plants (*31k*), in which the expression of *U11/U12-31K* is reduced with an amiR-mediated knockdown strategy (Web MicroRNA Designer; http://wmd3.weigelworld.org), were described in our previous report (Kim *et al.*, 2010). To generate transgenic plants expressing QQT1, drought-induced 19-2 (Di19-2), or the E2FB transcription factor in the *31k* mutant background, the pCB302-3 vector carrying the cDNA encoding each protein under the control of the *Cauliflower mosaic virus* 35S promoter was introduced into the Arabidopsis *31k* mutant by vacuum infiltration (Bechtold and Pelletier, 1998) using *Agrobacterium tumefaciens* GV3101. To construct amiR-mediated *QQT1* knockdown plants (*qqt1*), amiRs with the target site in the first exon of *QQT1* were designed using the Web MicroRNA Designer. The pCB302-3 vector carrying the amiR gene was introduced into the wild-type Arabidopsis plant by vacuum infiltration, and the T_3_ or T_4_ homozygous lines were used for phenotypic analysis.

### Analysis of the splicing patterns of U12 intron-containing genes

For analysis of the splicing patterns of U12 intron-containing genes, total RNA was extracted from 20-day-old wild-type, *31k* mutant, *QQT1*-expressing *31k* mutant, and amiR-mediated *qqt1* mutant plants. The extracted RNAs were treated with RQ1 DNase (Promega, Madison, WI, USA) and purified using an RNeasy clean-up kit (Qiagen, Valencia, CA, USA). Splicing patterns of U12 introns were analyzed via reverse transcription–PCR (RT–PCR) as previously described (Kim *et al.*, 2010; Jung and Kang, 2014). Briefly, the pre-mRNAs and mature mRNAs were amplified using a one-step RT–PCR kit (Qiagen) with gene-specific primers (Kim *et al.*, 2010), and the PCR products were separated on a 1% agarose gel and visualized under UV light.

### RNA extraction, RT–PCR, and quantitative real-time RT–PCR

Total RNA was extracted from leaf samples using a GeneAll Hybrid-R™ kit (GeneAll Biotechnology Co. Ltd, Seoul, Korea), and the purity and concentration of the RNA were quantified using a NanoDrop US/ND-1000 spectrophotometer (Thermo Scientific, Waltham, MA, USA). For RT–PCR, 500 ng of total RNA was amplified using a one-step RT–PCR kit (Qiagen) with the gene-specific primers listed in Supplementary Table S1 at *JXB* online. For quantitative real-time RT–PCR, 100 ng of total RNA was amplified using a SYBR Green kit (Qiagen) with the gene-specific primers listed in Supplementary Table S1 in a Rotor-Gene Q real-time thermal cycling system (Qiagen). Actin was used as an internal control.

### Northern blot analysis

For the detection of 21-mer mature amiRs, 20 μg of total RNA was separated on a denaturing 12% polyacrylamide gel and transferred to a nylon membrane as previously described (Kim *et al.*, 2010). RNA blots were hybridized in 1× Ultrahyb-Oligo hybridization buffer (Ambion, Austin, TX, USA) with a radiolabeled probe complementary to the amiR. The membrane was washed with washing buffer (2× SSC, 0.25% SDS), and the amiR signals were detected using a phosphorimager (GE Healthcare Life Sciences, Pittsburgh, PA, USA).

### Yeast two-hybrid screening

Yeast two-hybrid screening was performed essentially as described previously (Kim *et al.*, 1997). For the construction of the bait plasmid, the full-length *QQT1* cDNA was cloned into the pBD-GAL4 CAM vector (Stratagene, La Jolla, CA, USA). Both the bait plasmid and cDNA library clones were transformed into the yeast strain Y190 (Clontech, Mountain View, CA, USA). A total of 1 × 10^6^ transformants were screened for growth on synthetic defined (SD) agar medium depleted of leucine, tryptophan, and histidine (LTH), and supplemented with 50 mM 3-amino-1,2,4-triazole. The QQT1-interacting proteins were selected after further screening by an X-gal filter lift assay for β-galactosidase activity.

### Firefly LCI assay

The cDNAs encoding full-length TOC33, PSBS, PSAG, LHCB1, LHCA2, and RBCS2B were subcloned into the pDONRzeo vector using Gateway^®^ BP Clonase™ II Enzyme mix (Invitrogen, Carlsbad, CA, USA), and then transferred to the pCAMBIA1300-nLUC or pCAMBIA1300-cLUC vector (Chen *et al.*, 2008; Supplementary Fig. S1). The LCI assay was performed as described previously (Chen *et al.*, 2008). Briefly, the *Agrobacterium* strains containing each construct and the p19 strain were infiltrated into the leaves of 5-week-old *Nicotiana benthamiana,* and the plants were grown in the growth room for 2 d. Immediately before observing the images, 1 mM luciferin was sprayed onto the leaves, and the leaves were kept in the dark for 5 min to quench the fluorescence. The LUC images were observed with a G:BOX Chemi XL system (Syngene, Frederick, MD, USA) at 5 min intervals.

### Chloroplast protein extraction and immunoblot analysis

For the isolation of chloroplasts, 10 g of plant tissue was ground in 40 ml of cold extraction buffer (50 mM HEPES-KOH, pH 7.9, 330 mM d-sorbitol, 1 mM MgCl_2_, 2 mM EDTA, and 0.1% BSA). After filtering through eight layers of gauze, 14 ml of Percoll medium (50 mM HEPES-KOH, pH 7.9, 40% Percoll, 300 mM d-sorbitol, and 0.1% BSA) was added into the filtrates. After centrifugation at 3220 *g* for 5 min, the pellets were washed twice with the extraction buffer, and suspended in 2 ml of the suspension buffer [10 mM HEPES-KOH, pH 7.9, 4 mM MgCl_2_, and 1 mM phenylmethylsulfonyl fluoride (PMSF)]. The chloroplast proteins were extracted by adding a homogenization buffer (50 mM Tris–HCl, pH 7.5, 1 mM EDTA, 250 mM sucrose, 100 mM DTT, 5 mM leupeptin, and 100 mM PMSF). Approximately 10 μg of extracted protein was separated via 12% SDS–PAGE and transferred to a polyvinylidene fluoride membrane. The primary antibodies specific to each protein and the horseradish peroxidase-conjugated secondary antibody were purchased from Agrisera (Vännäs, Sweden). The signals were detected with an Amersham chemiluminescence kit (GE Healthcare Life Sciences), and the intensity of each band was quantified using a Gene Snap and Gene Tools software (Syngene).

## Results

### *QQT1* rescues the developmental-defect phenotypes of the *U11/U12-31K* mutant

**Fig. 1.**
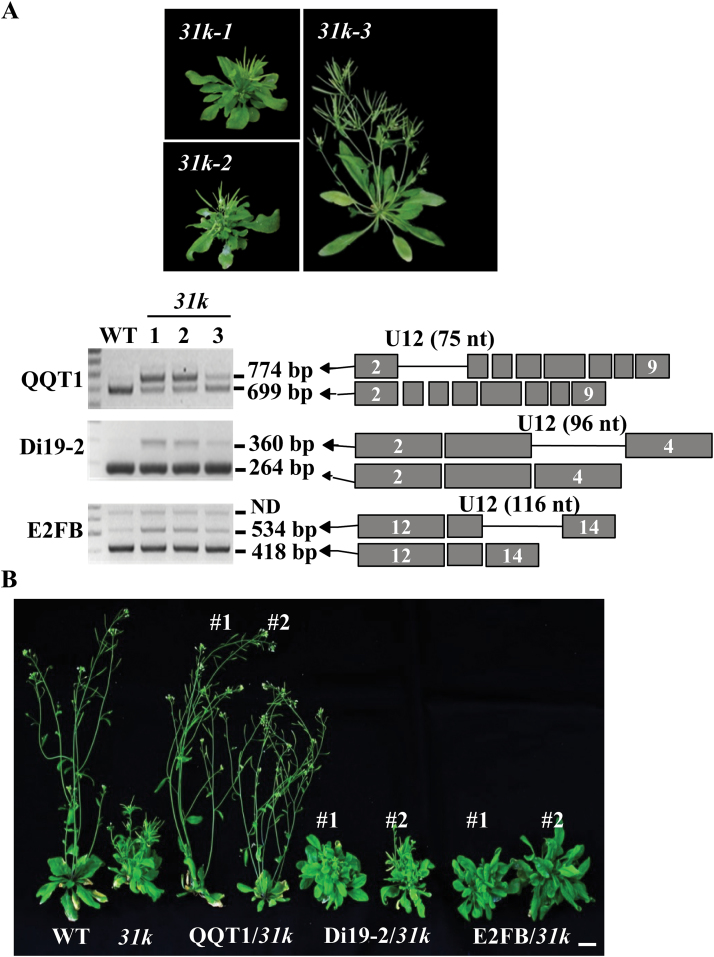
Phenotypes of QQT1-, Di19-2-, or E2FB-expressing U11/U12-31K mutants. (A) Splicing of *QQT1*, *Dil19-2*, and *E2FB* was analyzed in the artificial miRNA-mediated U11/U12-31K knockdown mutants (*31k-1*, *31k-2*, and *31k-3*). (B) The gene encoding QQT1, Di19-2, or E2FB was introduced in the *31k* mutant background, and phenotypes of the two independent transgenic plants (#1 and #2) expressing each gene are shown. Scale bar=1 cm. (This figure is available in colour at *JXB* online.)

Although our previous study showed that developmental defects of the *31k* mutant plant were caused by improper splicing of many U12 introns (Kim *et al.*, 2010), the key question of which U12 intron-containing genes are essential for plant development has not yet been explored. To determine which U12 intron-containing genes are responsible for the observed developmental-defect phenotypes of the *31k* mutant plant, the splicing patterns of the U12 intron genes were comprehensively analyzed in different mutant lines. Importantly, we found that the severity of the developmental defects in the *31k* mutant was closely correlated with the degree of defect in U12 intron splicing. In particular, the splicing of *QQT1* was closely correlated with the severity of the abnormal phenotypes of the *31k* mutant plant (Fig. 1A), suggesting that *QQT1* may have an important contribution to Arabidopsis development. To test this possibility, we generated transgenic Arabidopsis plants that expressed *QQT1* in the *31k* mutant background. We also generated transgenic Arabidopsis plants that expressed *Di19-2* or *E2FB* in the *31k* mutant background. Because the splicing of these two U12 intron-containing genes was not significantly altered in the *31k* mutant (Fig. 1A), we selected these genes as controls. Expression of each gene in transgenic plants was confirmed by RT–PCR analysis (Supplementary Fig. S2). As expected, the expression of *QQT1* rescued the developmental-defect phenotypes of the *31k* mutant to the wild-type phenotypes, whereas the expression of *Di19-2* or *E2FB* did not rescue the developmental defects of the *31k* mutant (Fig. 1B). These results suggest that the malfunction of QQT1 is the main factor contributing to the developmental defects observed in the *31k* mutant plant.

### QQT1 is essential for the normal development of Arabidopsis

**Fig. 2.**
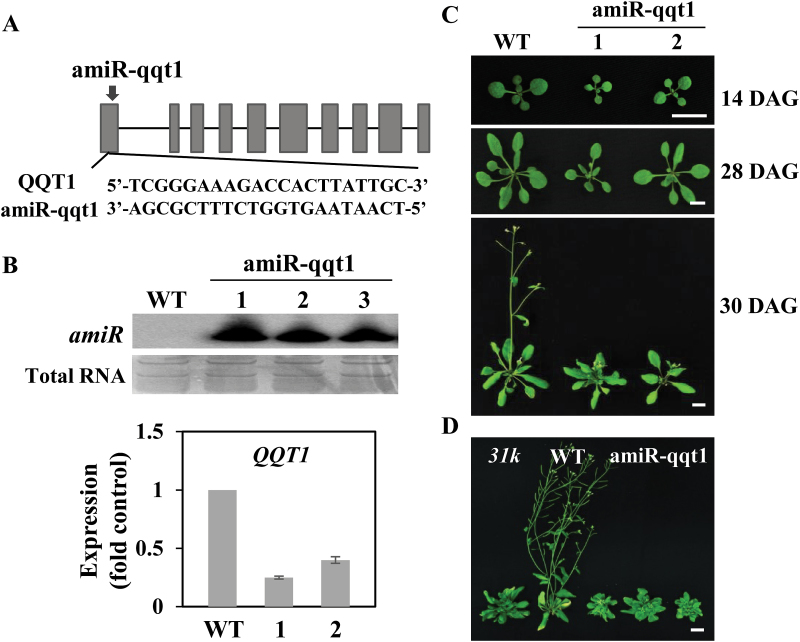
Generation and phenotype analysis of artificial miRNA-mediated *QQT1* knockdown plants. (A) Position of the artificial miRNA (amiR) target site and the sequences of amiR, along with its target, *QQT1*. Exons and introns are represented as gray boxes and thin lines, respectively. (B) Expression of the 21 nucleotide long mature *amiR* in each transgenic line (1, 2, and 3) was confirmed by northern blotting, and down-regulation of *QQT1* in the transgenic plants was confirmed by real-time RT–PCR analysis. (C) Growth-defect phenotypes of the *qqt1* mutant plants. Photographs of the wild-type (WT) and amiR-mediated mutant (1 and 2) plants were taken on the indicated days after germination (DAG). Scale bar=1 cm. (D) Comparison of phenotypes between the *qqt1* and the *31k* mutants. Scale bar=1 cm. (This figure is available in colour at *JXB* online.)

Because the *qqt1* knockout mutant embryo is lethal (Lahmy *et al.*, 2007), making it impossible to analyze its growth- and development-related phenotypes, we generated *qqt1* knockdown mutant plants using an amiR-mediated knockdown strategy as described previously (Schwab *et al.*, 2006; Kim *et al.*, 2010). The amiR lines targeting the first exon in *QQT1* were generated (Fig. 2A), and the production of a mature 21 nucleotide long amiR in the transgenic plants was confirmed by northern blot analysis (Fig. 2B). The transcript levels of *QQT1* in the amiR lines were ~25–40% of the wild-type level (Fig. 2B), confirming that the amiR lines are indeed knockdown mutants of *QQT1*. The *qqt1* mutant plants exhibited severe defects in growth and development, such as a small size, formation of serrated leaves, and arrested growth of primary inflorescence stems (Fig. 2C). Notably, the developmental-defect phenotypes of the *qqt1* mutant were quite similar to those of the *31k* mutant (Fig. 2D).

**Fig. 3.**
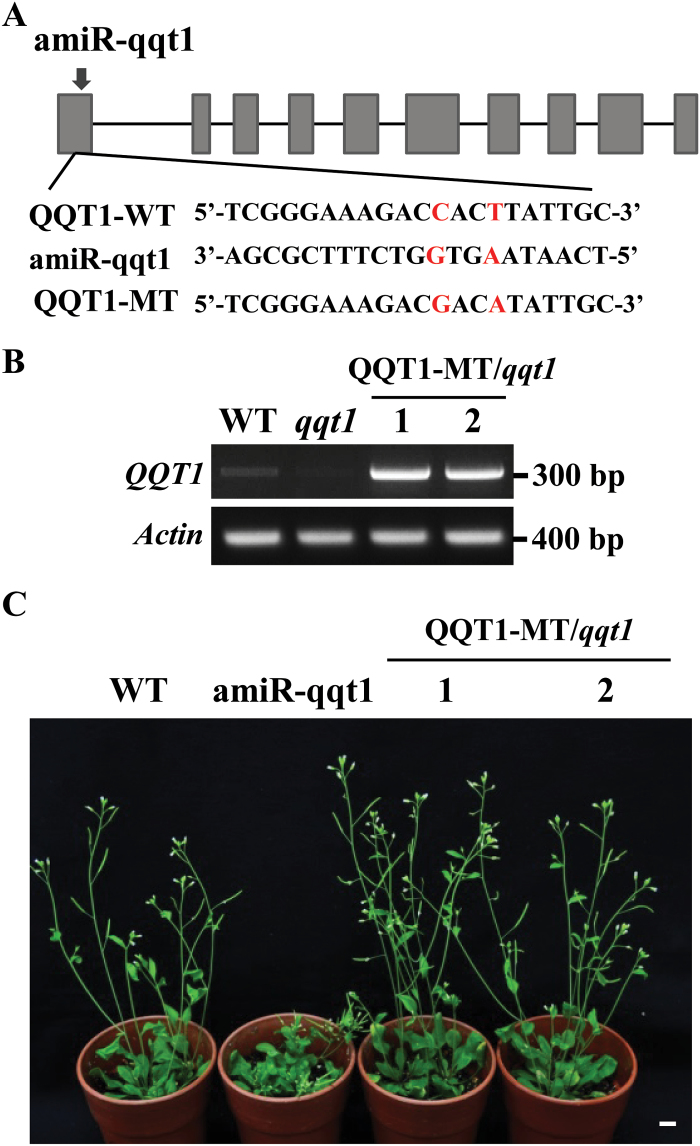
Recovery of normal phenotype in the complementation lines. (A) Schematic representation and sequences of artificial miRNA (amiR-qqt1) along with the wild-type QQT1 (QQT1-WT) and mutant QQT1 (QQT1-MT) designed to have G**·**G and A**·**A mismatches. (B) Absence of *QQT1* expression in the *qqt1* mutant and expression of *QQT1-MT* in the *qqt1* background in the complementation lines (1 and 2) were confirmed by RT–PCR analysis. (C) Photographs of the *qqt1* mutant (amiR-qqt1) and complementation lines expressing QQT1-MT (1 and 2) were taken 5 weeks after germination. Scale bar=1 cm. (This figure is available in colour at *JXB* online.)

To confirm further that specific knockdown of the *QQT1* gene is actually responsible for the developmental defects observed in the *qqt1* mutant plant, we generated complementation lines that expressed the mutant *QQT1* in the *qqt1* mutant background, which was designed to have C-to-G and T-to-A substitutions in the amiR target site (Fig. 3A), resulting in disruption of *QQT1* cleavage by the amiR. RT–PCR analysis showed that the transcript levels of *QQT1* in the complementation lines were much higher than those in the wild type and *qqt1* mutant (Fig. 3B), verifying the successful expression of the amiR-resistant *QQT1* in the complementation lines. The complementation lines displayed normal phenotypes that were comparable with those of the wild-type plant (Fig. 3C). These results further confirmed that QQT1 plays an essential role in the normal development of Arabidopsis.

**Fig. 4.**
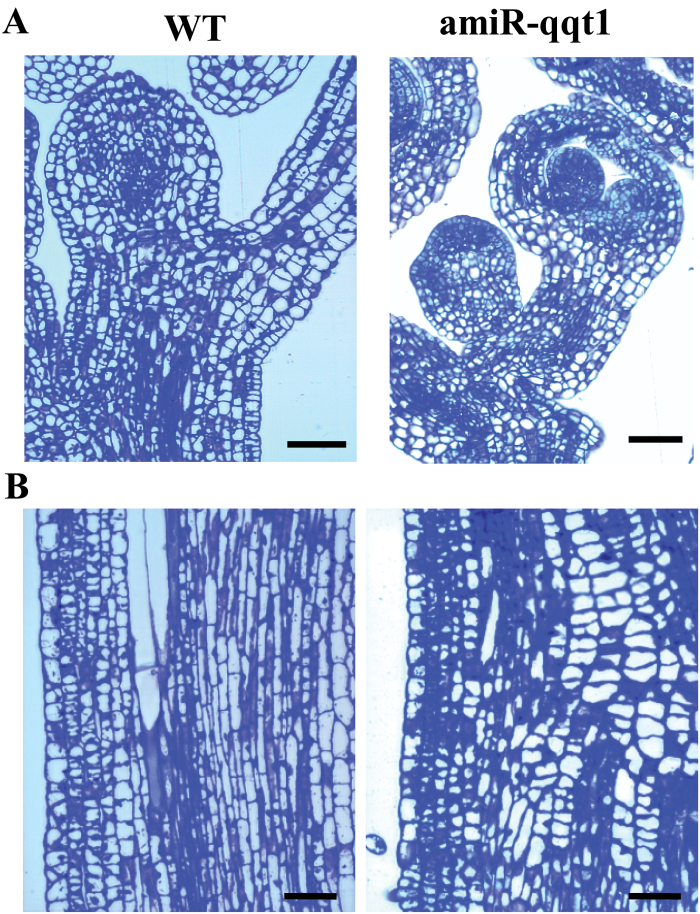
Images of the shoot apical meristem and stem regions of the wild-type and mutant plants. Longitudinal sections of the (A) shoot apical meristems and (B) stems of the 4-week-old wild-type (WT) and *qqt1* knockdown mutant (amiR-qqt1). Scale bar=50 μm. (This figure is available in colour at *JXB* online.)

To understand the cellular role of QQT1 in regulating plant development, the morphology of the vegetative shoot apical meristem (SAM) and the inflorescence stems was examined in 4-week-old wild-type and *qqt1* knockdown mutant plants. The results showed that the structures and organization of the SAM in the *qqt1* mutants were abnormal compared with those in the wild-type plants (Fig. 4A), and the cell shape and cell division activity of the inflorescence stems was significantly altered in the *qqt1* mutant (Fig. 4B). These results indicate that QQT1 plays a crucial role in Arabidopsis development via regulating cell division in the inflorescence stems. To examine the effect of exogenously applied hormones on the growth of the *qqt1* mutant, the *qqt1* mutant plant was treated with hormones known to influence stem growth and development, including GA_3_, cytokinin (kinetin), auxin (α-naphthalene acetic acid), and BR (24-epibrassinolide). None of the exogenous hormones rescued the developmental-defect phenotypes of the *qqt1* mutant (Supplementary Fig. S3).

### QQT1 does not affect the splicing of U12 introns

**Fig. 5.**
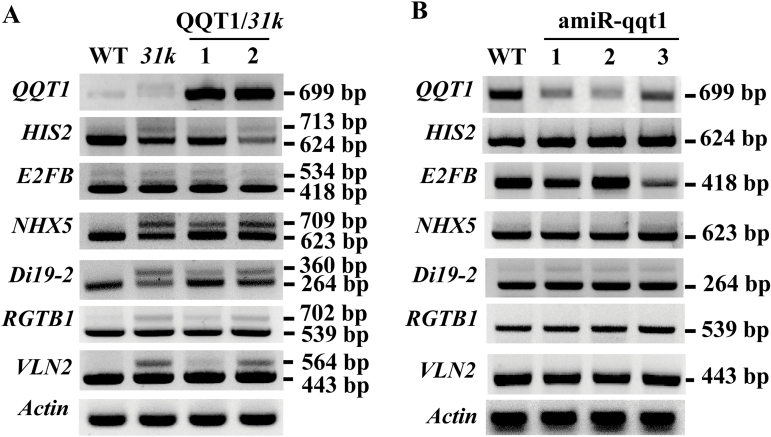
Splicing patterns of U12-type introns in the wild-type and mutant plants. Splicing patterns of several U12 intron-containing transcripts were analyzed by RT–PCR in the (A) wild-type (WT), U11/U12-31K knockdown mutant (*31k*), and QQT1-expressing *31k* mutants (1 and 2) and (B) wild-type (WT) and *QQT1* knockdown mutant (amiR-qqt1; 1, 2, and 3). Identical results were obtained from three independent experiments, one of which is shown. The sizes (bp) of unspliced (upper band) and spliced (lower band) products of each gene are indicated on the right.

Given that the phenotypes of the *qqt1* mutant were quite similar to those of the *31k* mutant, whose developmental-defect phenotypes are caused by U12-type intron splicing defects (Kim *et al.*, 2010), we next sought to determine whether QQT1 affects the splicing of U12 introns. To this end, we analyzed the splicing patterns of U12 introns in the wild-type and mutant plants. Among the U12 intron-containing genes in Arabidopsis, we focused on genes that are conserved across organisms with annotated putative functions, and their splicing patterns were analyzed by RT–PCR as described previously (Kim *et al.*, 2010; Jung and Kang, 2014). The results showed that expression of *QQT1* in the *31k* mutant background did not restore the normal splicing of U12 introns in the *31k* mutant (Fig. 5A; Supplementary Fig. S4A). Moreover, none of the U12 introns showed altered splicing in the *qqt1* mutant (Fig. 5B; Supplementary Fig. S4B). These results suggest that the developmental defects observed in the *qqt1* mutant are not due to defects in U12 intron splicing, indicating that QQT1 does not affect the splicing of U12 introns.

### QQT1 interacts with a variety of proteins

**Fig. 6.**
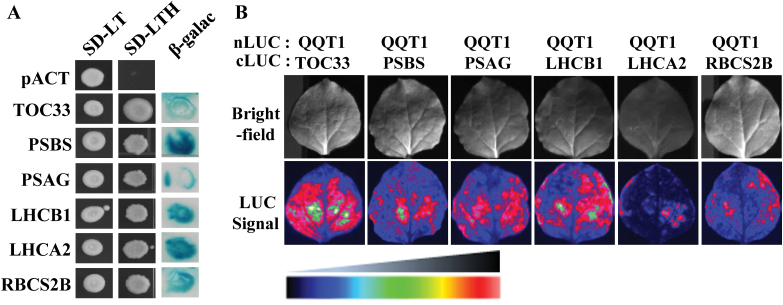
Interactions between QQT1 and various cellular proteins. (A) Yeast two-hybrid assay. Positive interactors were selected based on the growth in –Leu/Trp/His media (SD-LTH) and β-galactosidase activity. (B) Luciferase (LUC) complementation imaging assay. The luciferase images of tobacco leaves co-infiltrated with the N-terminal LUC-QQT1- and the C-terminal LUC-interacting proteins were observed with a chemiluminescent imager. (This figure is available in colour at *JXB* online.)

Because QQT1 plays an essential role in plant development, a remaining important question is to determine the cellular role of QQT1 during plant development. Given that QQT is an ATP/GTP-binding protein that is co-localized with the microtubules (Lahmy *et al.*, 2007), it is likely that QQT1 exerts its role by interacting with other proteins. To test this hypothesis, a yeast two-hybrid screen was performed to search for candidate proteins that interact with QQT1. A cDNA library of Arabidopsis seedlings was used as the prey, and the full-length QQT1 was used as bait. After screening 3 × 10^8^ clones, 49 positive clones were selected, and sequencing analysis of the positive clones ultimately identified 26 clones (Fig. 6A; Supplementary Fig. S5; Supplementary Table S2). Interestingly, 14 out of the 26 clones were predicted to be localized to the chloroplast. To confirm further the interactions between QQT1 and the putative QQT1-interacting proteins (QIPs) *in planta*, a firefly LCI assay was performed in tobacco (*N. benthamiana*) leaves. For the LCI assay, the QQT1 and candidate proteins were fused to the N- and C-terminal fragments of luciferase (LUC), respectively, and the constructs were transiently co-expressed in tobacco leaves. Strong LUC signals were observed in the positive control pair (SGT1b–RAR1) and no LUC signals were observed in the negative control pair (QQT1–QQT1) (Supplementary Fig. S6). To confirm further the absence of LUC signals in the negative control pairs, interactions between QQT1 and several unrelated proteins, including the U11/U12-31K, -59K, and -65K proteins that are localized to the nucleus and are involved in the splicing of U12 introns (Will *et al.*, 2004; Kim *et al.*, 2010; Jung and Kang, 2014), were analyzed. In addition, the GRP7 protein that is localized to the cytoplasm and the nucleus and is involved in stress response (Kim *et al.*, 2008) was also analyzed. Clearly, no LUC signals were observed in these negative control pairs (Supplementary Fig. S6). Among the 26 genes identified with yeast two-hybrid screening, 7 representative genes were subjected to the LCI assay. The results showed that the tobacco leaves co-transformed with the QQT1–nLUC and QIPs–cLUC constructs exhibited strong fluorescence signals (Fig. 6B), confirming the interactions between QQT1 and these QIPs in plants. The identified QIPs include the PSI subunit G (PSAG), PSII subunit S (PSBS), PSI light-harvesting complex gene 2 (LHCA2), PSII light-harvesting complex gene B1B1 (LHCB1), translocon at the outer envelope membrane of chloroplasts 33 (TOC33), Rubisco small subunit 2 (RBCS2B), and microtubule-associated protein 65-7 (MAP65-7).

### QQT1 is involved in chloroplast biogenesis and affects the level of chloroplast proteins

**Fig. 7.**
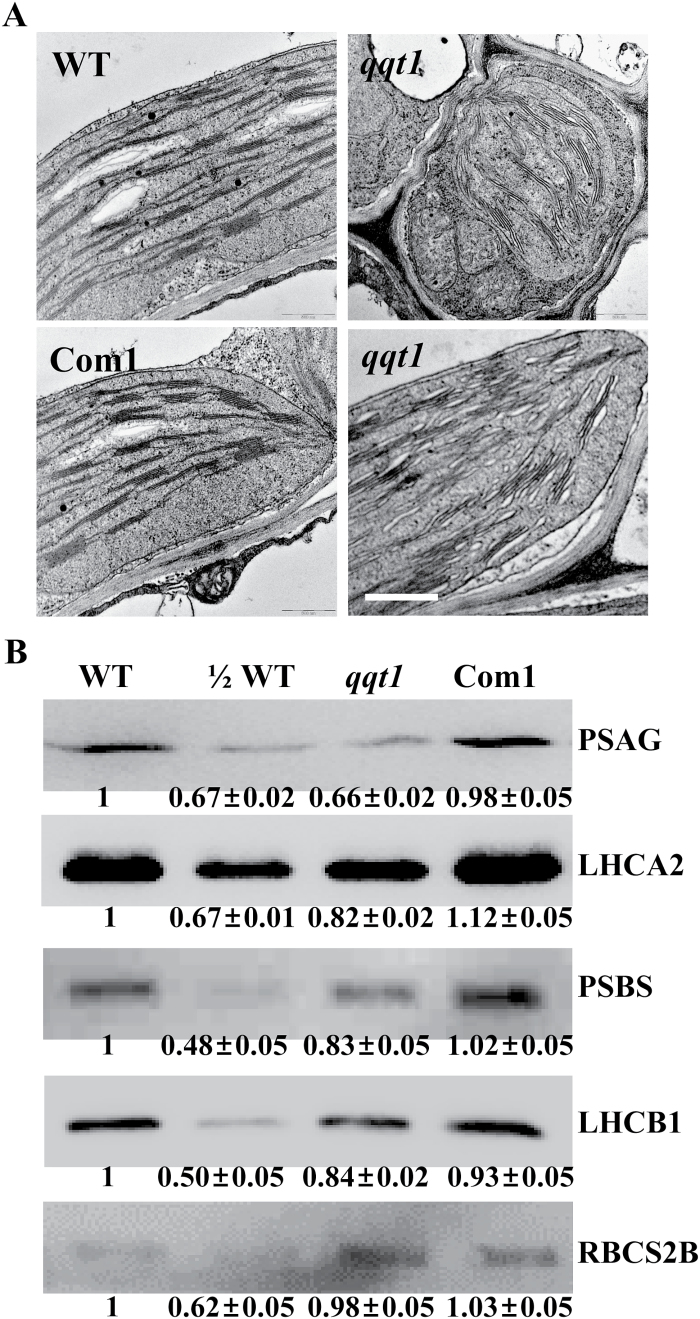
Chloroplast structures and analysis of the chloroplast proteins in the wild-type and mutant plants. (A) Chloroplast structures of 4-week-old wild-type (WT), *QQT1* knockdown mutant (*qqt1*), and complementation line (Com1) were observed using transmission electron microscopy. Scale bar=20 nm. (B) Immunoblot analysis of the levels of QQT1-interacting proteins. Chloroplast proteins (10 µg) were separated by SDS–PAGE, and the proteins were detected using an antibody specific to each protein; ‘½ WT’ indicates that half the amount of chloroplast protein (5 µg) was loaded in the lane. The intensity of each band was quantified using a Gene Snap and Gene Tools software, and the values are the means ±SE of three independent analyses.

Given that QQT1 clearly interacts with many chloroplast-targeted proteins, it is likely that it is involved in the transport of these proteins to the chloroplast, which is important for chloroplast biogenesis and function. To test this hypothesis, the structures of the chloroplasts in the wild-type and *qqt1* mutant plants were examined by transmission electron microscopy. The results showed that the wild-type plants maintained intact and regular grana lamellae of the thylakoid membrane, whereas the *qqt1* mutants had fewer stacked thylakoids and exhibited a disturbed thylakoid membrane organization (Fig. 7A). This abnormal chloroplast structure found in the *qqt1* mutant recovered to the wild-type structure in the complementation line (Fig. 7A). We next analyzed the level of chloroplast proteins that were identified as QIPs. Immunoblotting analysis of the protein fractions obtained from the isolated chloroplasts revealed that the levels of PSAG, PSBS, LHCA2, and LHCB1 in the *qqt1* mutant were ~66–84% of the wild-type level, whereas the levels of these proteins in the complementation line were comparable with those in the wild-type plant (Fig. 7B).

## Discussion

The results of our study revealed that QQT1 is a crucial factor for the normal development of Arabidopsis. Several reports demonstrated that U11/U12-31K, U11-48K, and U11/U12-65K mutants displayed severe developmental-defect phenotypes, and most of their U12 intron-containing genes showed altered splicing (Kim *et al.*, 2010; Jung and Kang, 2014; Xu *et al.*, 2016). These previous findings suggested that the cumulative defects in U12 intron splicing are responsible for the abnormal developmental phenotypes observed in the *31k*, *48k*, and *65k* mutant plants. However, a firm link between the developmental defects and abnormal splicing of a specific U12 intron in plants has not been demonstrated. Many U12 intron-containing genes are involved in seed maturation and seedling growth, pollen tube guidance and embryo patterning, flowering time regulation, and the light signaling pathway (Milla *et al.*, 2006; Tsukagoshi *et al.*, 2007; Li *et al.*, 2011; Ana *et al.*, 2012). A critical remaining question is to determine the specific U12 intron-containing genes that are responsible for the abnormal developmental phenotypes observed in the U11/U12-31K, U11-48K, and U11/U12-65K mutant plants. Our complementation analysis of QQT1 in the *31k* mutant background (Fig. 1) and the analysis of amiR-mediated *qqt1* knockdown mutants (Figs 2, 3) clearly demonstrated that among the >200 U12 intron-containing genes in Arabidopsis, QQT1 plays a crucial role in the normal development of Arabidopsis. A previous report showed that a knockout mutant of *QQT1* resulted in a lethal embryo (Lahmy *et al.*, 2007), implying that QQT1 is essential for early embryo development. Our sequence analysis of QQT1 from diverse plant species showed that QQT1 proteins in dicot and monocot plants share ~80–90% amino acid sequence homology (Supplementary Fig. S7), suggesting that QQT1 is a highly conserved protein that plays an essential role in plant development. A higher expression level of *QQT1* in the SAM than in other tissues (https://apps.araport.org/thalemine) and the abnormal structures and shapes of the SAM and cells in the inflorescence stems of the *qqt1* mutant plant (Fig. 4) suggest that QQT1 affects plant development by regulating meristem activity and cell division activity.

The most important remaining question is to determine how QQT1 affects plant development. A hint of this mechanism comes from the fact that QQT, an ATP/GTP-binding protein, is co-localized with the microtubules in Arabidopsis (Lahmy *et al.*, 2007). The microtubule is a cytoskeleton component found throughout the cytoplasm and provides platforms for intracellular transport (Karki and Holzbaur, 1999; Vale, 1987, 2003). In this study, a yeast two-hybrid screen and LCI assay were employed to identify potential proteins that interact with QQT1. Among the many QQT1-interacting proteins were those targeted to chloroplasts, including TOC33, ATP-PRT1, RBCS2B, RBCS3B, PSAG, LHCA2, LHCB1, and PSBS, and nuclear proteins, such as EPR1, RAP2.12, and RAD23C (Fig. 6; Supplementary Fig. S5; Supplementary Table S2). Notably, the majority of the QIPs identified in this study were chloroplast-targeted proteins (Supplementary Table S2). A variety of chloroplast proteins are encoded in the nucleus and are then transported to chloroplasts, during which the TOC (translocon at the outer envelope membrane of chloroplasts) and TIC (translocon at the inner envelope membrane of chloroplasts) complexes in the chloroplast co-operate to drive the post-translational import of the proteins across the chloroplast membranes (Soll and Schleiff, 2004; Smith, 2006; Inaba and Schnell, 2008; Jarvis, 2008). The importance of the chloroplast targeting of proteins has been demonstrated in the analysis of Arabidopsis *plastid protein import 1* (*ppi1*) and *ppi2* mutants, which lack TOC33 and TOC159, respectively, that displayed albino phenotypes due to deficiencies in the import of photosynthesis-related proteins (Jarvis *et al.*, 1998; Kubis *et al.*, 2003; Smith *et al.*, 2004). Importantly, QQT1 interacted with the TOC33 protein (Fig. 6), suggesting that QQT1 is involved in the transport of the chloroplast-targeted proteins into the chloroplast. Notably, our results showed that the levels of chloroplast proteins, such as PSAG, LHCA2, LHCB1, and PSBS, were noticeably decreased in the *qqt1* mutant (Fig. 7B), suggesting that malfunction of QQT1 affects the transport of many chloroplast proteins and chloroplast biogenesis. Evidently, the structures of the chloroplast in the *qqt1* mutant were found to be abnormal with a disturbed thylakoid membrane organization (Fig. 7A). Collectively, our results suggest that QQT1 participates in the transport of many proteins to chloroplasts, which is essential for chloroplast biogenesis and function and for the normal development of Arabidopsis plants. It would be interesting to determine how the mutation in *QQT1* affecting chloroplast transport of many proteins is related to embryo-defective phenotypes with embryos arrested at the octant stage as observed in a previous study (Lahmy *et al.*, 2007). In addition, because QQT1 interacts with several proteins localized to the nucleus, membrane, or cytosol (Supplementary Table S2), the physiological significances of these interactions should be explored to understand fully the mechanistic role of QQT1 in plant development.

In conclusion, the present results demonstrate that *QQT1* is an indispensable U12 intron-containing gene, whose splicing is crucial for the normal development of *A. thaliana*. Considering that most chloroplast proteins are encoded in the nucleus and are then transported to chloroplasts, our finding that QQT1 interacts with many chloroplast-targeting proteins provides a valuable basis for further investigation of the QQT1-mediated transport of chloroplast proteins. It would be of interest to determine further how QQT1 specifically recognizes its interacting partners and ultimately influences the transport of chloroplast-targeting proteins.

## Supplementary data

Supplementary data are available at *JXB* online.

Fig. S1. Schematic diagrams of 35S::NLuc and 35S::CLuc constructs.

Fig. S2. Confirmation of the expression of target genes in transgenic plants.

Fig. S3. Effects of exogenously applied hormones on the growth of the *qqt1* knockdown mutant plants.

Fig. S4. Splicing patterns of U12-type introns in the wild-type and mutant plants.

Fig. S5. Yeast two-hybrid assay showing the interactions between QQT1 and various cellular proteins.

Fig. S6. Luciferase (LUC) complementation imaging assay.

Fig. S7. Alignment of the amino acid sequences of QQT1 proteins from various plant species.

Table S1. Gene-specific primers used in RT–PCR and real-time RT–PCR analysis.

Table S2. List of the putative QQT1-interacting proteins identified by a yeast two-hybrid screening.

## Supplementary Material

supplementary_tables_S1_S2_figures_S1_S7
