# Supplementary material for: quatre-quart1 is an indispensable U12 intron-containing gene that plays a crucial role in Arabidopsis development
Source: J Exp Bot. 2017 May 5;68(11):2731–9. doi: 10.1093/jxb/erx138 (PMC5853960; doi:10.1093/jxb/erx138)
Supplement: supplementary_tables_S1_S2_figures_S1_S7 [file erx138_suppl_supplementary_tables_s1_s2_figures_s1_s7.pdf]

Supplementary Table S1. Gene-specific primers used in RT-PCR and real-time RT-PCR analysis

Gene	Primer (5' to 3')
	For real-time RT-PCR
QQT1	Forward: ATGGTGTTTGGACAAGTAG
	Reverse: GTAATGATCCTTCAGAAGAG
Actin	Forward: CAGCAGAGCGGGAAATTGTAAGAG
	Reverse: TTCCTTTCAGGTGGTGCAACGAC
	For RT-PCR
QQT1	Forward: ATGGTGTTTGGACAAGTAGTA
	Reverse: TCAGTCTTGTATTTCCTCATCTTC
Di19-2	Forward: CGATATAGCAGTGGAGTATCCATGC
	Reverse: GGCAACTCATCAAGATGAGGTTGAAG
E2FB	Forward: GTTCCTGATGAACCCTCTAATGTCC
	Reverse: GTGATCTCGTAGCAGTGGATTCTTC
Actin	Forward: TGCGATAATGGAACTGGTATGG
	Reverse: AAGACAGCCCTGGGCGCATCA

Supplementary Table S2. List of the putative QQT1-interacting proteins identified by a yeast two-hybrid screening.

Gene	Localization	Description
AT1G02280	chloroplast	ATTOC33, PLASTID PROTEIN IMPORT 1, PPI1, TOC33, TRANSLOCON AT THE OUTER ENVELOPE MEMBRANE OF CHLOROPLASTS 33
AT1G04985	mitochondrion	unknown protein
AT1G14690	nucleus	MAP65-7, MICROTUBULE-ASSOCIATED PROTEIN 65-7
AT1G18330	nucleus	EARLY-PHYTOCHROME-RESPONSIVE1, EPR1, REVEILLE 7, RVE7
AT1G27770	membrane/chloroplsat, ER, plasma	ACA1, AUTOINHIBITED CA2+-ATPASE 1, PEA1, PLASTID ENVELOPE ATPASE 1
AT1G44575	chloroplast	CP22, NONPHOTOCHEMICAL QUENCHING 4, NPQ4, PHOTOSYSTEM II SUBUNIT S, PSBS
AT1G53910	nucleus, membrane	RAP2.12, RELATED TO AP2 12
AT1G55670	chloroplast	PHOTOSYSTEM I SUBUNIT G, PSAG
AT1G57990	chloroplast	ATPUP18, PUP18, PURINE PERMEASE 18
AT1G58080	chloroplast	ATATP-PRT1, ATP PHOSPHORIBOSYL TRANSFERASE 1, ATP-PRT1, HISN1A
AT2G28320	nucleus, membrane	Pleckstrin homology (PH) and lipid-binding START domains-containing protein
AT2G34420	chloroplast	LHB1B2, LHCB1.5, PHOTOSYSTEM II LIGHT HARVESTING COMPLEX GENE 1.5, PHOTOSYSTEM II LIGHT HARVESTING COMPLEX GENE B1B2
AT3G02540	nucleus	PUTATIVE DNA REPAIR PROTEIN RAD23-3, RAD23-3, RAD23C, RADIATION SENSITIVE23C
AT3G17440	chloroplast, plasma membrane	ATNPSN13, NOVEL PLANT SNARE 13, NPSN13
AT3G48860	chloroplast	SCD2, STOMATAL CYTOKINESIS DEFECTIVE 2
AT3G55980	nucleus	ATSZF1, SALT-INDUCIBLE ZINC FINGER

		1, SZF1
AT3G61470	chloroplast	LHCA2, PHOTOSYSTEM I LIGHT HARVESTING COMPLEX GENE 2
AT4G17730	membrane, nucleus	ATSYP23, SYNTAXIN OF PLANTS 23, SYP23
AT4G21830	chloroplast, cytosol	ATMSRB7, METHIONINE SULFOXIDE REDUCTASE B7, MSRB7
AT4G28040	chloroplast, membrane	UMAMIT33, USUALLY MULTIPLE ACIDS MOVE IN AND OUT TRANSPORTERS 33
AT5G07980	nucleus, plasmodesmata	dentin sialophosphoprotein-related
AT5G15350	anthored to membrane	ATENODL17, EARLY NODULIN-LIKE PROTEIN 17, ENODL17
AT5G19510	apoplast, cytosol	Translation elongation factor EF1B/ribosomal protein S6 family protein
AT5G22920	nucleus	CHY-type/CTCHY-type/RING-type Zinc finger protein
AT5G38410	apoplast, cell wall, chloroplast	RBCS3B, RUBISCO SMALL SUBUNIT 3B
AT5G38420	apoplast, chloroplast	RBCS2B, RUBISCO SMALL SUBUNIT 2B

Supplementary Figure S1. Schematic diagrams of 35S::NLuc and 35S::CLuc constructs. QIP, QQT-interacting protein; 35S, cauliflower mosaic virus 35S promoter; rbs, transcription terminator derived from the rubisco small subunit gene.

Supplementary Figure S2. Confirmation of the expression of target genes in transgenic plants. Overexpression of each gene in independent transgenic plants (#1 and #2) was confirmed by RT-PCR analysis.

Supplementary Figure S3. Effects of exogenously applied hormones on the growth of the *qqt1* knockdown mutant plants. Phenotypes of the plants 2 weeks after application of 100 µM GA, 50 µM kinetin, 5 µM BR, or 0.5 µg/ml α-naphthalene acetic acid (NAA)

Supplementary Figure S4. Splicing patterns of U12-type introns in the wild-type and mutant plants. Splicing patterns of several U12 intron-containing transcripts were analyzed by RT-PCR in the (A) wild-type (WT), U11/U12-31K knockdown mutant (*31k*), and QQT1-expressing *31k* mutants (1 and 2) and (B) wild-type (WT) and QQT1 knockdown mutant (amiR-qqt1; 1, 2, and 3). Identical results were obtained from three independent experiments, one of which is shown. The sizes (base pair) of un-spliced (upper band) and spliced (lower band) products of each gene are indicated at right.

Supplementary Figure S5. Yeast two-hybrid assay showing the interactions between QQT1 and various cellular proteins. Positive interactors were selected in –Leu/Trp/His media (SD-LTH), and filter lift assays were conducted for monitoring expression of the *LacZ* reporter gene (β-galactosidase).

Supplementary Figure S6. Luciferase (LUC) complementation imaging assay. The N-terminal LUC-target protein and the C-terminal LUC-target protein were co-infiltrated in *N. benthamiana* leaves, and the luciferase images of each leaf were observed under a fluorescence microscope. SGT1b and RAR1 were used as positive controls, and several unrelated proteins, including U11/U12-31K, -59K, -65K, and GRP7, were used as a negative control.

Supplementary Figure S7. Alignment of the amino acid sequences of QQT1 proteins from various plant species. The alignment was made using a ClustalW program. The accession numbers for each protein are as follow; *Arabidopsis thaliana* (AT5G22370), *Triticum aestivum* (CDM83967), *Zea mays* (NP_001131405), *Oryza sativa* (XP_015626052), *Glycine max* (XP_003523622), *Solanum tuberosum* (XP_006356856), *Capsicum annuum* (XP_016568874), *Camelina sativa* (XP_010493318), and *Brassica rapa* (XP_009144235).

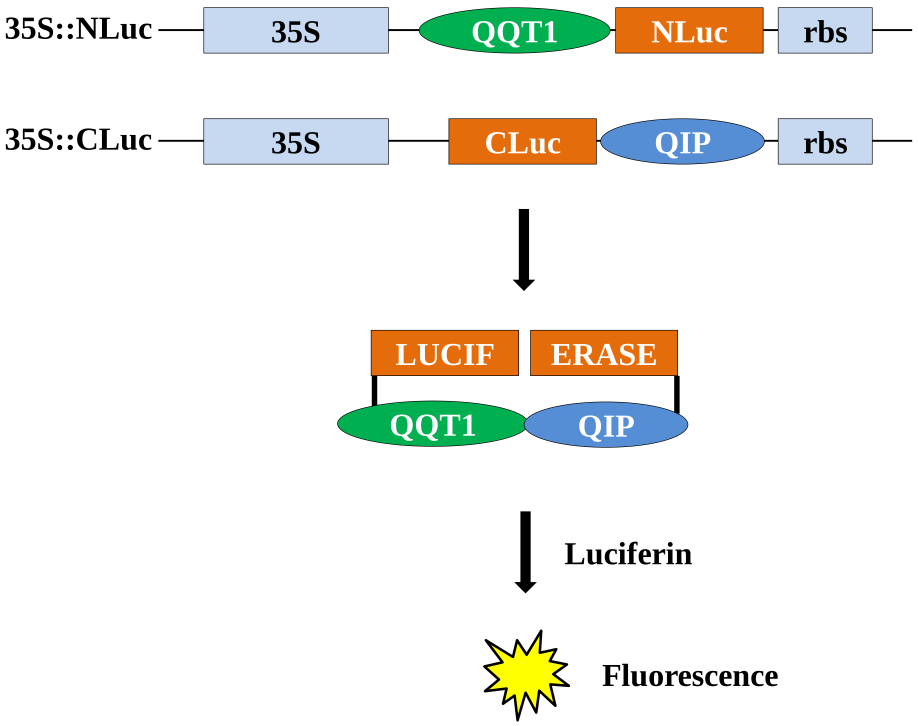

Supplementary Figure S1

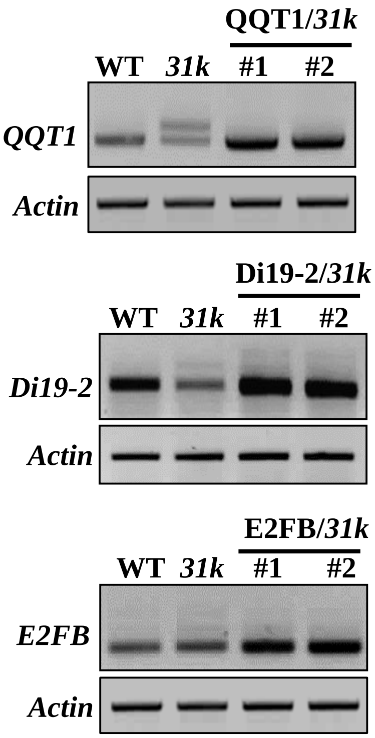

Supplementary Figure S2

Mock +GA +Kinetin +BR +NAA

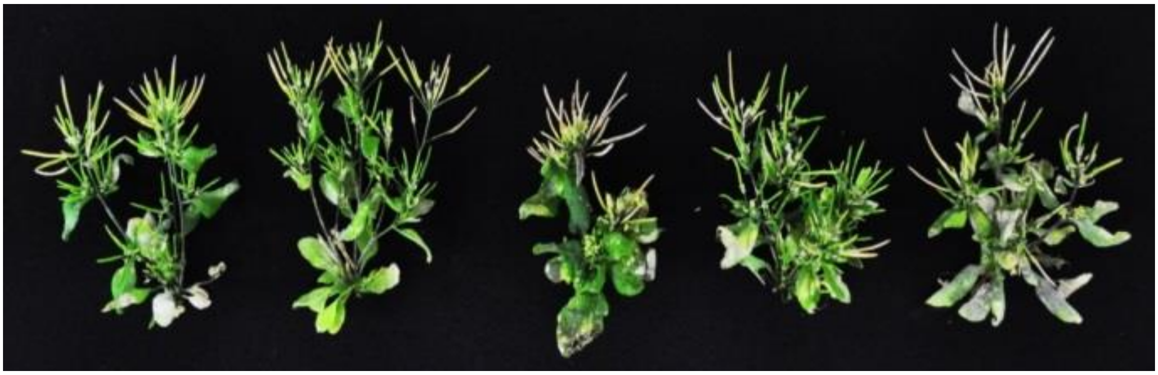

Supplementary Figure S3

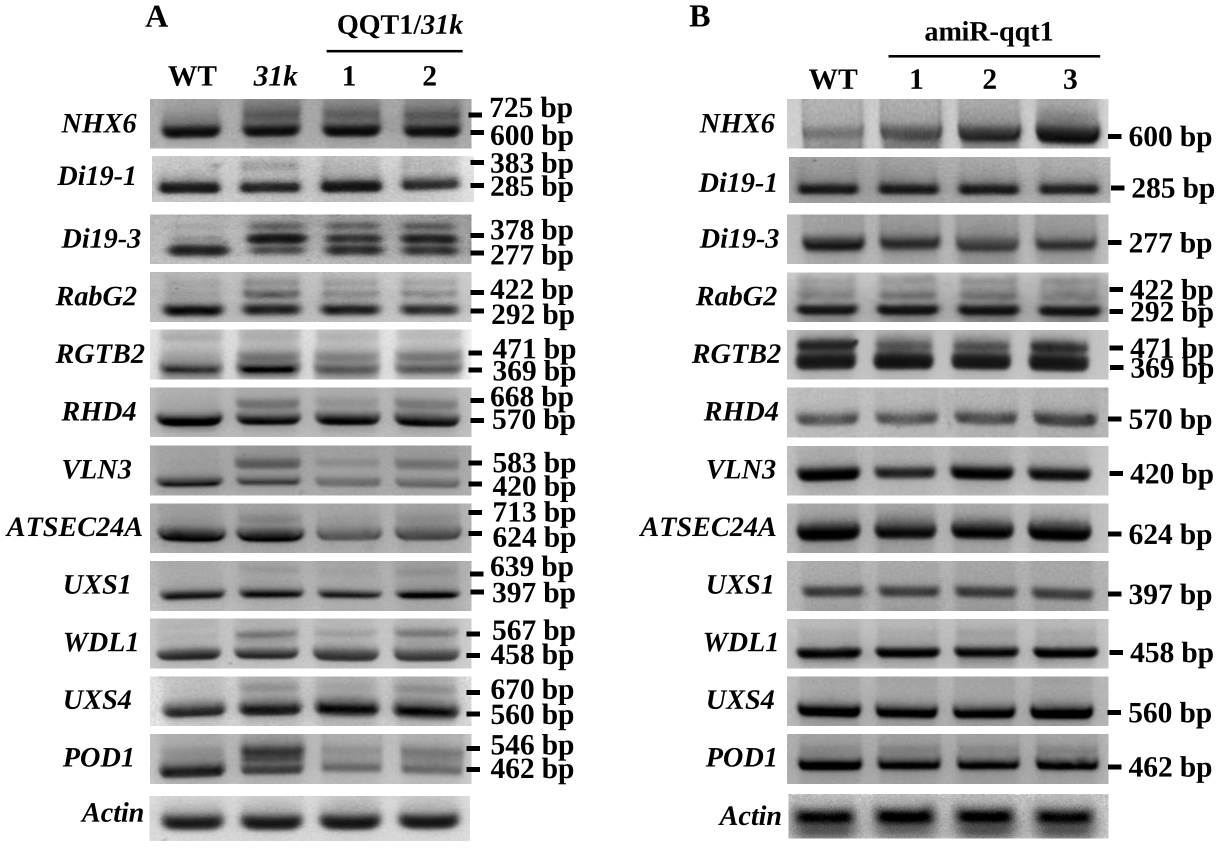

Supplementary Figure S4

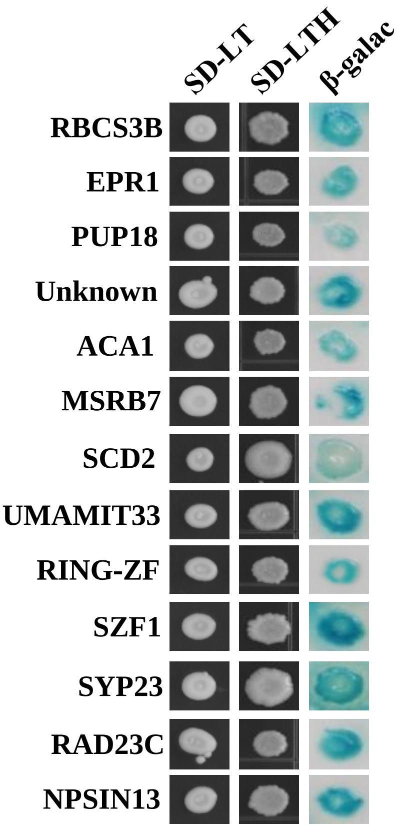

Supplementary Figure S5

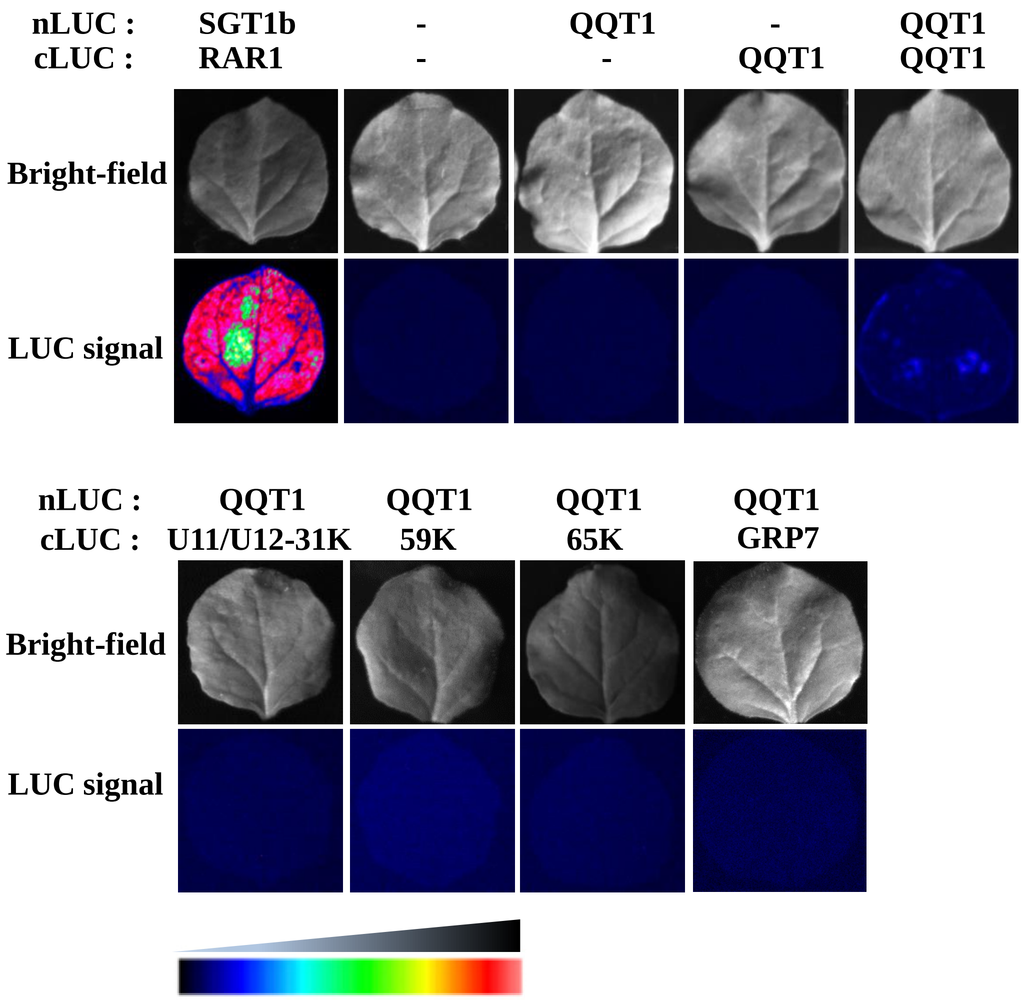

Supplementary Figure S6

```
A.thaliana  1 MVFGQVVIGPPGSGKTTYCNGMSQFLSLMGRKVAIVNLDPANDALPYECGVNIEELIKLEDVMSEHSLGP
T.aestivum  1 MVFGQVVIGPPGSGKTTYCNGMSQFLSLVGRKVAVINLDPANDALPYECAINIEDLIKLSDVMSEHSLGP
Z.mays      1 MVFGQVVIGPPGSGKTTYCNGMSQFLSLLGRKVAVVNLDPANDALPYECAINIEDLIKLSDVMSEHSLGP
O.sativa    1 MVFGQVVIGPPGSGKTTYCNGMSQFLSLVGRKVAVINLDPANDVLPYECAINIEDLIKLSDVMSEHSLGP
G.max       1 MVFGQVVVGPPGSGKTTYCNGMSQFLSLIGRKVAVINLDPANDSLPYECAVNIEDLVKLSDVMVEHSLGP
S.tuberosum 1 MVFGQVVIGPPGSGKTTYCNGMSQFLQLIGRKVAVINLDPANDALPYECAVNIEDLIKLSDVMVEHSLGP
C.annuum    1 MVFGQVVIGPPGSGKTTYCNGMSQFLQLIGRKVAVINLDPANDALPYECAVNIEDLIKLSDVMVEHSLGP
C.sativa    1 MVFGQVVIGPPGSGKTTYCNGMSQFLPLIGRKVAIVNLDPANDALPYECAVNIEDLIKLEDVMLEHSLGP
B.rapa      1 MVFGQIVIGPPGSGKTTYCNGMSQFLSLIGRKVAVVNLDPANDALPYECAVNIEELIKLEVVMAECSLGP

A.thaliana 71 NGGLVYCMEYLEKNIDWLESKLKPLLKDHYILFDFPGQVELFFIHDSTKNVLTKLIKSLNLRLTAVQLID
T.aestivum 71 NGGLVYCMDYLEMNIDWLEEKLKPLIEDHYFLFDFPGQVELFSLHTNARNIINKLIKKLDLRLTAVHLVD
Z.mays     71 NGGLVYCMDYLEKNIDWLEEKLKPFIEDHYLLFDFPGQVELFFLHSNARSVINKLIKKMDLRLTAVHLID
O.sativa   71 NGGLVYCMDYLEKNIDWLEEKLKPLIEDHYLLFDFPGQVELFFLHSNARSIIYKLIKKLNLRLTAVHLID
G.max      71 NGGLVYCMDYLEKNIDWLEAKLEPLLKDHYLLFDFPGQVELFFLHSSAKNVILKLIKKLNLRLTAVHLID
S.tuberosum71 NGGLVYCMDYLEKNIDWLESKLKPLLKEHYLLFDFPGQVELFFLHDNAKNMIMELIKKLDLRLTAVHLVD
C.annuum   71 NGGLVYCMDYLMKNIDWLESRLKPLLKEHYLLFDFPGQVELFFLHENAKNMIMELIKKLDLRLTAVHLVD
C.sativa   71 NGGLVYCMEYLEKNIDWLESKLKPLLKDHYILFDFPGQVELFFIHNSTKNVLTKLIKSLNLRLTAVQLID
B.rapa     71 NGGLLHCMEYLEKNIDWLEAKLKPLSKDHYFLFDFPGQVELFFNHDSTKKVLTKLTKSLNLRLTAVQLID

A.thaliana141 SHLCCDPGNYVSSLLLSLSTMLHMELPHVNVLSKIDLIGSYGKLAFNLDFYTDVQDLSYLEHHLSQDPRS
T.aestivum141 AHLCCDPGKYVSALLLSLSTMLHLELPHINVLSKIDLIENYGNLAFNLDFYTDVQDLSYLQYHLDQDPRS
Z.mays    141 AHLCCDPGKYVSALLLSLSTMLHLELPHINVLSKIDLIENYGNLAFNLDFYTDVQDLSYLQYHLEQDPRS
O.sativa  141 AHLCCDPGKYVSALLLSLSTMLHMELPHINVLSKIDLIENYGNLAFNLNFYTDVEDLSYLQRHLDQDPRS
G.max     141 AHLCSDPGKYISALILSLSTMLHLELPHINVLSKIDLIESYGKLAFNLDFYTDVQDLSYLQHHLDQDPRS
S.tuberosu141 AHLCSDPGKYVSALLLSLSTMLHLELPHVNVLSKIDLIESYGKLAFNLDFYTDVQDLSYLQNEISQDPRS
C.annuum  141 AHLCSDPGKYVSALLLSLSTMLHLELPHVNVLSKIDLIESYGKLAFNLDFYTDVQDLSYLQNALSQDPRS
C.sativa  141 AHLCSDPGNYVSALLLSLSTMLHMELPHVNVLSKIDLIGSYGKLAFNLDFYTDVQDLSYLEHHLGQDPRS
B.rapa    141 SVLCTDPGNYVSALLLSLSTMLHMELPHINVLSKIDLIGNYGKLAFGLDFYTDVQDLSYLQNYLNQDPRS

A.thaliana211 AKYRKLTKELCSVIEDYSLVNFTTLDIQDKESVGDLVKLIDKSNGYIFAGIDASVVEYSKIAIGQTDWDY
T.aestivum211 AKYRKLTKELCDVIDDFSLVNFSTLDIQDKESVGNLVKLIDKSNGYIFSSIDSSAVEFSKIAAAPLDWDY
Z.mays    211 AKYRKLTKELCDVIDDFGLVNFSTLDIQDKESVGNLVKLIDKSNGYIFSSIDSSAVEFSKIAAAPLDWDY
O.sativa  211 AKYRKLTKELCDVIDDFGLVNFTTLDIQDKESVGNLVKLIDKSNGYIFSSIDSSVVEFSKIAAAPLDWDY
G.max     211 AKYRKLTKELCDIIENFSLVSFSTLDIQDKESVGNLVKLIDKSNGYIFVGMEASAVEFSKIAVGPVNWDY
S.tuberosu211 AKYRKLTKELCEVIEDYGLVNFTPLDIQDKESVGNLVKLIDKSNGYIFAGIDASAVEFSKIAVGPVDWDY
C.annuum  211 AKYRKLTKELCEVIEDYGLVNFTTLDIQDKESVGNLVKLIDKSNGYIFAGIDASAVGFSKIAVGPVDWDY
C.sativa  211 AKYRKLTKELCSVIEDFSLVNFTTLDIQDKESVGNLVKLIDKSNGYIFSGIDASVVEYSKIAVGQTDWDY
B.rapa    211 VKYRKLTGELCSVVEDYGLVSFTTLDIQDKESVGELVKLIDKSNGYIFAGIDASVVEYSKISVRQTDWEY

A.thaliana281 NRVAAVQEKYMEDEEIQD--------
T.aestivum281 YRTAAVQEKYMKDDEIVQKTSGMQ--
Z.mays    281 YRTAEVQEKYMKDDEFAQLTSRMQ--
O.sativa  281 YRTAEVQEKYMKDDEFVQKTSKTR--
G.max     281 YRVAAVQEKYMKDDENIDDNE-----
S.tuberosu281 YRVAAVQEKYIKDDEDVDMTKDSETK
C.annuum  281 YRVAAVQEKYIKDDEDFDMTRNSDTK
C.sativa  281 NRVAAVQEKYMNDEETQD--------
B.rapa    281 NRVAAVQEKYMKDEDTED--------
```

Supplementary Figure S7
